# Outcomes of total elbow arthroplasty in trauma patients compared with patients following fixation of distal humerus fractures

**DOI:** 10.1016/j.jor.2025.04.013

**Published:** 2025-05-06

**Authors:** David Maman, Itay Ron, Nada Weishahe, Yaniv Keren, Mitchell Yelton, Yaniv Trior, Elad Apt, Bezalel Peskin, Nabil Ghrayeb, Doron Norman, Jacob Shapira

**Affiliations:** a The Ruth and Bruce Rappaport Faculty of Medicine, Technion - Israel Institute of Technology, Haifa, Israel; b Orthopedic Department, Rambam Medical Center, Haifa, Israel; c Michigan State University College of Human Medicine, East Lansing, MI, USA

**Keywords:** Total elbow arthroplasty, Distal humerus fractures, Patient reported outcomes, Open reduction internal fixation of distal humerus

## Abstract

**Background and purpose:**

Total elbow arthroplasties (TEA) aim to replicate anatomy and provide stability in the treatment of distal fractures of the humerus. In the presence of an aging population with higher functional demand, improving patients' well-being is crucial. This study aimed to analyze patients’ reported outcomes and functional outcomes for TEA in comminuted fractures of the distal humerus and to compare these outcomes with their counterpart patients who have been treated with open reduction and internal fixation (ORIF). In addition, this study aims to compare the secondary procedures rate between the two groups.

**Patients and methods:**

Eligible patients were those who underwent TEA or ORIF of the distal humerus and completed several patient-reported outcome (PRO) questionnaires including the Disabilities of the Arm, Shoulder, and Hand (DASH) questionnaire, 12-Item Short Form Health Survey Physical and Mental components (SF-12 P and SF-12 M, respectively) scores, visual analog scale (VAS) score for pain, and patient satisfaction ratings (1–5). A physical examination including range of motion, instability, and strength was performed for all patients.

**Results:**

This study found that patients following TEA have shown significantly lower pain levels (TEA: 3.72 ± 2.8; ORIF 5.2 ± 2.98, P = 0.019) and higher satisfaction levels (TEA: 4.18 ± 1.17; ORIF 3.57 ± 1.46, P = 0.035) compared with patients following ORIF. DASH score (TEA: 33.7 ± 29.4; ORIF 39.75 ± 24.6, P = 0.31) and SF-12 score (TEA: 31.7 ± 9.67; ORIF 31.25 ± 10.2, P = 0.85) were not statistically different between TEA and ORIF.

Patients following TEA demonstrated an advantage in flexion in the operative arm compared with patients following ORIF (P = 0.045). Both patients following TEA and ORIF demonstrated no statistically significant difference in protonation and supination of the operated elbow compared to the contralateral side. Although, a decreased range in extension and flexion of the operated elbow compared with the contralateral side was demonstrated in both groups (extension P = 0.005, flexion P < 0.001). The Grip Test showed no significant difference between the patients who were treated by TEA or ORIF (P = 0.99). Moreover, ORIF in comminuted fractures of the distal humerus in elders may be associated with a higher complication rate compared with TEA.

**Conclusion:**

TEA following comminuted fractures of the distal humerus is associated with favorable satisfaction, pain levels, and range of motion in flexion compared with patients following ORIF of the distal humerus. Additionally, TEA may be associated with a lower rate of secondary procedures.

## 1 Introduction

Intra-articular fractures of the distal humerus are considered a complex injury that necessitates high surgical skills to restore proper function of the injured limb. Overall, fractures of the distal humerus constitute approximately five percent of osteoporotic fractures in patients over 60 years old.^1^ Since the function of the elbow depends on the faultless architecture of the articular surface as well as the preserved mechanical axis of the joint, most of these fractures are managed with open reduction and internal fixation (ORIF) to achieve anatomical reduction and stable fixation.^2^

Due to poor bone quality and excessive damage to the periarticular soft tissue in the elderly, thriving for anatomical reduction and stable fixation is not always feasible.^3^^,^^4^ Moreover, the healing process of intra-articular fractures involving both columns of the distal humerus may be halted since fragments are mostly covered with cartilage and are thus deprived of sufficient soft tissue and blood supply.^5^ These inherent drawbacks in intra-articular fractures of the distal humerus may lead to poor outcomes including functional limitation and decreased range of motion, non-union, and consequently, a relatively high rate of secondary surgeries.^6^^,^^7^ As high as 20 % of patients who have sustained distal humeral fractures have demonstrated either loss of function, dissatisfaction, or sub functional range of motion.^8^

In 1997, Cobb and Murray proposed total elbow arthroplasty (TEA) as an alternative repair option for comminuted fractures of the humerus in the elderly and patients with end-stage arthritis of the elbow.^9^ The body of literature is ample with studies for TEA in rheumatic patients, whereas, despite the increasing popularity of TEA in trauma, evidence is still relatively scarce.^5^ One literature review demonstrated some gravitation towards a better range of motion and satisfaction in elderly patients who have undergone TEA compared with ORIF, however, there is no consensus for the optimal management of comminuted fractures of the distal humerus in this patient population.^10^^,^^11^

In the presence of an aging population with higher functional demand, improving patients' well-being is crucial. Considering the potential derangement involving comminuted fractures of the distal humerus, it is of great importance to investigate the outcomes following TEA in elderly trauma patients. Therefore, the aims of this study were: (1) to analyze the patients’ reported outcomes for TEA in comminuted fractures of the distal humerus in elders, (2) to compare these outcomes with their counterpart patients who have been treated with ORIF, and (3) to compare the complications rate and secondary procedures between the groups.

## 2 Methods

### 2.1 The study population

This is a prospectively collected and retrospectively reviewed study which includes patients who were treated surgically for intra-articular fractures of the distal humerus between the years 2003–2020. The study contains 88 patients: the study group contains 47 patients who were treated by TEA and the control group contains 41 patients who were treated by ORIF.

All patients were admitted and operated in a tertiary trauma center, in an orthopedic department with a specific shoulder and elbow unit.

During this study's relevant years several kinds of implants were used for TEA, mainly of 2 companies (Stryker, Zimmer Biomet).

Inclusion criteria: patients aged 18 years and older who had distal humeral fractures treated surgically.

Exclusion criteria: patients were excluded if they had previous elbow conditions, such as rheumatic disease or neoplastic disease.

During the initial admission to the hospital, each patient was examined, each patient's specific treatment was decided after a thorough medical examination of an orthopedic surgeon which specializes in elbow injuries. The main considerations taken into account as part of the decision included the patient's baseline functional and activity level, the severity of the fracture, age, and the patient's preference after being informed about the treatment options.

### 2.2 Patient reported Outcome questionnaires scoring

Patients completed several patient-reported outcome (PRO) questionnaires including the Disabilities of the Arm, Shoulder and Hand (DASH) questionnaire, 12-Item Short Form Health Survey Physical and Mental components (SF-12 P and SF-12 M, respectively) scores, visual analog scale (VAS) score for pain (1–10 where 10 is most painful), and patient satisfaction ratings (1–5 where 5 is most satisfied).

The DASH questionnaire is suitable for patients with upper extremity musculoskeletal conditions and consists of 30 items. Each item is scored on a 5-point scale. Twelve questions assess activities of daily living, 6 questions assess symptoms, and 3 questions assess social and work limitations. The scores for all items are used to calculate a final score ranging from 0 (no disability) to 100 (severe disability). Reliability, validity, and responsiveness of the DASH have been evaluated in patients with disorders of all major areas of the extremity, i.e., shoulder, elbow, wrist, and hand.^12^

The 12- item Short Form Survey (SF-12) is a health-related quality-of-life questionnaire consisting of twelve questions that measure eight health domains to assess physical and mental health. Physical health-related domains include General Health (GH), Physical Functioning (PF), Role Physical (RP), and Body Pain (BP). Mental health-related scales include Vitality (VT), Social Functioning (SF), Role Emotional (RE), and Mental Health (MH).^13^ Higher scores indicate better physical and mental health functioning.

Moreover, complications and secondary procedures, such as revision arthroscopy or conversion to TEA, were also documented.

### 2.3 Physical examination

A physical examination was performed during follow-up visits for all patients. Range of motion, instability, and strength were evaluated for all patients. Strength was evaluated with the Grip Test and reported as the average of 3 measurements.

### 2.4 Statistical analysis

Descriptive statistics in terms of mean, standard deviation, median, percentiles, and ranges were calculated for the whole parameters in the study. The normal distribution of continuous parameters was tested by the Shapiro-Wilk test. Parametric or non-parametric tests were then calculated accordingly. Differences between the two groups (TEA vs. ORIF) according to patient age, VAS, satisfactions, SF-12 M, SF-12 P, and Dash score were calculated by *t*-test or Mann Whitney *U* test. For categorical parameters, we used the Fisher exact test. Paired tests were used for the difference of several parameters (flexion, extension, protonation, and supination) between the surgery elbow vs. healthy elbow. Repeated measure analysis was calculated for differences in flexion, extension, protonation, and supination between surgery elbow vs. healthy elbow and between the two groups (TEA vs. ORIF). P value < .05 was considered as significant. SPSS software version 28 was used for statistical analysis.

## 3 Results

### 3.1 Patient selection and demographics

Overall, 88 eligible patients met the inclusion and exclusion criteria. Patients were distributed into 2 groups: the study group - TEA - included 47 patients (53.4 %) and the control group - ORIF - included 41 patients (47.6 %). The mean age in the study group was 74 ± 10.1 years with the youngest patient being 45 years old and the oldest patient being 91 years old. There were 6 males (13 %) and 41 females (87 %) in the study group. In the control group, the mean age was 56.4 ± 17 years with the youngest patient being 23 years old and the oldest patient being 79 years old and included 25 males (61 %) and 16 females (39 %). The TEA group was statistically significantly older and included more females than the ORIF group in this study. The average follow-up period was 42 months [Range: 23.8–82.0] in the TEA group and 46.5 month [Range: 29.5–73.5] in the ORIF group (P = 0.86) (Table 1).

**Table 1:** Patient demographics.

	Study group TEA; n = 47	Control group ORIF; n = 41	P-value
Age (Mean ± SD)	74.3 ± 10.10	56.4 ± 17.00	**P<0.001**

Gender		N = 35	**P<0.001**
Female	41 (87 %)	16 (39 %)	
Male	06 (13 %)	25 (61 %)	
Laterality		N = 40	P = 0.200
Left	29 (62 %)	19 (47.5 %)	
Right	18 (38 %)	21 (52.5 %)	
Dominant Side		N = 40	P = 0.560
Left	05 (11 %)	03 (7.5 %)	
Right	41 (87 %)	37 (92.5 %)	
Both	01 (2 %)	00	
Follow Up (Mean [Range])	42 [23.80–82.00]	46.50 [29.50–73.50]	P = 0.860

n: sample size; SD: standard deviation; TEA: total elbow arthroplasty; ORIF: open reduction internal fixation.

### 3.2 Patients reported outcomes

The assessment tools included the DASH score, SF-12 M and SF-12 P scores, VAS score, and satisfaction rate. Patients following TEA have shown significantly lower pain levels (TEA: 3.72 ± 2.8; ORIF 5.2 ± 2.98, P = 0.019) and higher satisfaction levels (TEA: 4.18 ± 1.17; ORIF 3.57 ± 1.46, P = 0.035) compared with patients following ORIF.

DASH score (TEA: 33.7 ± 29.4; ORIF 39.75 ± 24.6, P = 0.31) and SF-12 score (TEA: 31.7 ± 9.67; ORIF 31.25 ± 10.2, P = 0.85) were not statistically different between TEA and ORIF (Table 2).

**Table 2:** Patient reported outcomes.

	Study group TEA; n = 47	Control group ORIF; n = 41	P-value
VAS (Mean ± SD)	3.72 ± 2.80	5.20 ± 2.98; n = 40	**P=0.019**
Satisfaction (Mean ± SD)	4.18 ± 1.17	3.57 ± 1.46	**P=0.035**
SF-12 -total out of 47 (Mean ± SD)	31.70 ± 9.67	31.25 ± 10.20	P = 0.850
SF-12 M (Mean ± SD)	17.90 ± 5.60	18.50 ± 6.20	P = 0.650
SF-12 (Mean ± SD)	13.67 ± 4.43	13.60 ± 4.22	P = 0.920
DASH Score (Mean ± SD)	33.70 ± 29.40	39.75 ± 24.60	P = 0.310

n: sample size; SD: standard deviation; TEA: total elbow arthroplasty; ORIF: open reduction internal fixation; VAS: visual analog scale for pain (range 1–10); Satisfaction (range 1–5); SF-12 M: 12 item short form survey mental health (out of 27); SF-12 P: 12 item short form survey physical health (out of 20); DASH: disabilities of the arm, shoulder, and hand questionnaire (range 0–100).

### 3.3 Physical examination

A physical examination was performed for the patients during follow-up visits. Measurements included flexion, extension, supination, and protonation. A statistical analysis was made to inspect the differences between the TEA and ORIF. Patients following TEA have demonstrated an advantage in flexion compared with patients following ORIF (P = 0.045, Table 3)

**Table 3:** Range of motion.

GROUP		Flexion – Operated elbow	Extension – Operated elbow	supination - Operated elbow	Pronation - Operated elbow
TEA	Mean	129.11	13.46	84.82	87.86
	N	28	28	28	28
	Std. Deviation	12.624	18.947	9.377	4.797
	Median	130.00	7.50	90.00	90.00
	Minimum	90	00	50	75
	Maximum	150	87	90	90
ORIF	Mean	119.64	11.07	87.86	87.14
	N	14	14	14	14
	Std. Deviation	16.463	7.385	8.018	8.254
	Median	120.00	10.00	90.00	90.00
	Minimum	70	0	60	60
	Maximum	140	30	90	90
Total	Mean	125.95	12.67	85.83	87.62
	N	42	42	42	42
	Std. Deviation	14.535	15.969	8.966	6.073
	Median	130.00	10.00	90.00	90.00
	Minimum	70	0	50	60
	Maximum	150	87	90	90
P-value		**P=0.045**	P = 0.650	P = 0.310	P = 0.720

N: sample size; TEA: total elbow arthroplasty; ORIF: open reduction internal fixation.

Another statistical analysis was made to compare the differences between the operated elbow and the contralateral elbow, separately for each TEA and ORIF. In both groups, measurements showed no significant difference between the two elbows in protonation or supination. However, significant differences were demonstrated for flexion and extension which showed a significant advantage to the contralateral elbow in both groups.

Patients undergoing a TEA had a significantly greater extension in their contralateral elbow compared to their operated elbow (P = 0.005, Table 4).

**Table 4:** Range of motion following TEA.

Descriptive Statistics									
	N	Mean	SD	Minimum	Maximum	Percentiles			
						25th	50th	75th	p-value
Flexion Operated Side	10	126.00	20.111	90	150	107.50	135.00	140.00	P = 0.120
Flexion Contralateral Side	10	137.50	5.401	125	140	137.50	140.00	140.00	
Extension Operated Side	10	20.70	26.107	5	87	5.00	7.50	28.75	**P=0.005**
Extension Contralateral Side	10	3.000	9.487	0	30	.00	.00	.00	
Supination Operated Side	10	87.50	6.346	70	90	88.75	90.00	90.00	P = 0.180
Supination Contralateral Side	10	90.00	.000	90	90	90.00	90.00	90.00	
Pronation Operated Side	10	90.00	.000	90	90	90.00	90.00	90.00	P = 1.000
Pronation Contralateral Side	10	90.00	.000	90	90	90.00	90.00	90.00	

N: sample size; TEA: total elbow arthroplasty; ORIF: open reduction internal fixation; SD: standard deviation.

Both patients following TEA and ORIF have demonstrated a significantly better range of motion in flexion of their contralateral side relative to the operated elbow (P < 0.001, Table 5). Furthermore, for Grip Test, we found no significant difference between the patients who were treated by TEA or ORIF (P = 0.99, Table 6)

**Table 5:** Comparing Range of motion following TEA and ORIF.

	GROUP	Mean	Std. Deviation	N	P = value
Flexion - Operated elbow	TEA	126.00	20.111	10	P = 0.400
	ORIF	119.64	16.463	14	
	Total	122.29	17.936	24	
Flexion - Contralateral elbow	TEA	137.50	5.401	10	P = 0.290
	ORIF	139.29	2.673	14	
	Total	138.54	4.032	24	

N: sample size; TEA: total elbow arthroplasty; ORIF: open reduction internal fixation.

p < 0.001.

**Table 6:** Grip test (average of 3).

Group	Mean	N	Std. Deviation	Median	Minimum	Maximum	P-value
TEA	25.306	17	13.7164	22.500	3.0	50.0	
ORIF	25.286	14	14.1553	21.000	11.0	65.0	
Total	25.297	31	13.6810	22.000	3.0	65.0	.990

N: sample size; TEA: total elbow arthroplasty; ORIF: open reduction internal fixation.

### 3.4 Secondary surgeries

In this study, 47 patients underwent TEA. Forty patients received a primary TEA procedure while seven patients needed a TEA as a secondary procedure after primary ORIF. The secondary TEA was indicated by limitation in the range of motion. Two patients out of forty who underwent a primary TEA required a secondary procedure after their primary TEA. One secondary procedure was due to wound dehiscence, and the other was because of postoperative stiffness (Table 7).

**Table 7:** – TEA group.

		Frequency	Percent	Valid Percent	Cumulative Percent
Valid	Primary TEA	40	85.1	85.1	85.1
	Primary ORIF	7	14.9	14.9	100.0
	Total	47	100.0	100.0	

## 4 Discussion

This study aimed to investigate outcomes following TEA due to fractures of the distal humerus. Patients following TEA demonstrated an advantage in flexion compared with patients following ORIF of the distal humerus (P = 0.045, Table 3). Of the reported range of motions, both patients following TEA and ORIF have demonstrated a decreased range in extension and flexion of the operated elbow compared with the contralateral side (extension P = 0.005, flexion P < 0.001; Table 4, Table 5). Additionally, patients following TEA reported less pain and higher satisfaction compared with patients following ORIF of the distal humerus. (Pain P = 0.019, satisfaction P = 0.035; Table 2). Of the patients following TEA, forty patients received a primary TEA procedure while seven patients needed a TEA as a secondary procedure after primary ORIF due to limitations in range of motion. Of the patients following primary TEA, two required a subsequent secondary procedure due to wound dehiscence and stiffness.

Patient-reported outcomes have been proposed as the true objective assessment tool for patients following orthopedic procedures.^14^^,^^15^ In a systemic review, Davey et al. aimed to evaluate the functional outcomes, dislocation, and revision rates following TEA at a minimum 10-year mean follow-up.^16^ Overall, 1276 patients following TEA from 23 studies were enrolled in their systematic review. Of these, 1060 patients had undergone TEA due to rheumatoid arthritis and 257 patients had undergone TEA due to other indications, including trauma. The average age was 64.7 years, and the mean follow-up was 137.2 months. At the final follow-up, the mean Mayo Elbow Performance (MEP) Score, Oxford Elbow Score, and Quick DASH scores were 89.1, 64.4, and 39.2, respectively. They indicated that 63.3 % of patients reported having no pain at follow-up. The authors concluded that TEA offers patients satisfactory clinical outcomes at long-term follow-up, with relatively stable revision and complication rates compared to short and mid-term follow-up. Corroborating with their results, this study found that patients following TEA are associated with better outcomes in terms of pain and satisfaction relative to patients following ORIF of the distal humerus. However, no significant difference was found in DASH and SF-12 scores between the two groups.

Additionally, this study aimed to investigate functional outcomes in patients following TEA. In their systemic review, Welsink et al. reported the results of the most performed TEAs. Overall, 9379 TEAs in seventy-three articles were enrolled in their study.^17^ The authors found that patients had a satisfactory reported range of motion at 6-year follow-up, with mean angles of flexion, extension, supination, and protonation of 129, 30, 66, and 71, respectively. The authors concluded that TEAs provide respectable functional outcomes at mid-term follow-up. These findings were also supported by Davey et al. in their long-term outcomes systemic review.^16^

The current study shows that patients following TEA demonstrate an advantage in flexion compared with patients following ORIF of the distal humerus. However, there was no statistically significant difference in grip strength between the patients who were treated by TEA or ORIF. Of the reported range of motions, both patient cohorts following TEA and ORIF demonstrated no statistically significant difference in protonation and supination of the operated elbow compared to the contralateral side. Although, a decreased range in extension and flexion of the operated elbow compared with the contralateral side was demonstrated (extension P = 0.005, flexion P < 0.001).

Secondary procedures are one of the foundations in assessing postoperative outcomes. In a multicenter prospective randomized trial, McKee et al. aimed to compare functional outcomes, complications, and reoperation rates in elderly patients with displaced intra-articular distal humeral fractures treated with either ORIF or primary TEA.^18^ Forty-two patients aged above 65 years old were randomized by a sealed envelope. The MEP score and DASH score were determined at 6 weeks, 3 months, 6 months, 12 months, and 2 years. Complication type, duration, management, and treatment requiring reoperation were recorded.

Reoperation rates for TEA (12 %) and ORIF (27 %) were not statistically different (P = 0.2). Since there was a non-significant trend towards a reduced reoperation rate in the TEA group, the authors concluded that TEA may result in decreased reoperation rates.

Accordingly, in the current study, seven patients underwent a subsequent TEA following ORIF due to debilitating limitations in range of motion. Of the patients following primary TEA, two patients had undergone secondary procedures due to stiffness and surgical wound dehiscence. These results have demonstrated that ORIF in comminuted fractures of the distal humerus in elders may be associated with a higher complication rate compared with TEA.

This study has a few limitations. First, both groups had a relatively low number of patients creating a low-powered study. Furthermore, there is selection bias related to age and gender, gravitating towards TEA in females and elders, due to the associated impairment in bone quality. Despite the older age of patients in the study group, patients following ORIF demonstrated less favorable pain and satisfaction. Lastly, the study was retrospectively analyzed leading to potential bias. However, to minimize potential bias, the data was still prospectively collected. Furthermore, due to its retrospective nature some of the data is missing, hence there is no sufficient information regarding complications and hardware loosening rates in both groups.

## 5 Conclusion

TEA following comminuted fractures of the distal humerus is associated with favorable satisfaction, pain levels, and range of motion in flexion compared with patients following ORIF of the distal humerus. Additionally, TEA may be associated with a lower rate of secondary procedures. Longer follow-up and further analysis of these factors will benefit clinical decision-making and understanding of patient indications for TEA versus ORIF.

## CRediT authorship contribution statement

**David Maman:** Writing – original draft. **Itay Ron:** Writing – review & editing, Project administration. **Nada Weishahe:** Data curation. **Yaniv Keren:** Investigation, Formal analysis. **Mitchell Yelton:** Writing – review & editing. **Yaniv Trior:** Investigation, Formal analysis. **Elad Apt:** Investigation, Formal analysis. **Bezalel Peskin:** Conceptualization, Supervision. **Nabil Ghrayeb:** Conceptualization, Supervision. **Doron Norman:** Conceptualization, Supervision. **Jacob Shapira:** Supervision, Writing – review & editing, Project administration, All authors have read and approved the final version of the manuscript.

## Informed consent statement

Informed consent was obtained from the patients.

## Ethics approval and consent to participate

All data collection and reporting received institutional review board approval.

## IRB approval English attest

This document is an English attest document to the attached IRB approval written in Hebrew.

Rambam medical center's Helsinki committee approved the conduct of medical study under the name " Outcomes of Total Elbow Arthroplasty in Trauma Patients Compared with Patients Following Fixation of Distal Humerus Fractures".

The approval was given by the institutional committee at January 11, 2022.

Chairman of the committee Prof. Shimon Pollak gave the approval to the main investigator Dr. Jacob Shapira.

## Funding

Not applicable.

## Declaration of competing interest

All authors report no conflict of interest regarding the preparation of this study. In addition, there was no funding to this research.
